# Creutzfeldt-Jakob disease mimicking Hashimoto’s encephalopathy: steroid response followed by decline

**DOI:** 10.1515/biol-2025-1245

**Published:** 2025-12-30

**Authors:** Tian-Chen Wu, Feng Zhao, Yan Liang, Zheng-Zheng Wu, Jin-Bin Chen, Zhen-Nian Zhang, Hui Yang

**Affiliations:** Department of Neurology, Nanjing Hospital of Chinese Medicine Affiliated to Nanjing University of Chinese Medicine, Nanjing 210000, China; School of Nursing, Nanjing University of Chinese Medicine, Nanjing 210000, China

**Keywords:** Creutzfeldt-Jakob disease, electroencephalogram, glucocorticoid pulse therapy, Hashimoto’s encephalopathy, magnetic resonance imaging

## Abstract

The diagnosis of Creutzfeldt–Jakob disease (CJD) is particularly challenging because its heterogeneous clinical presentations mimic other rapidly progressive dementias and neurodegenerative disorders (e.g., Hashimoto’s encephalopathy, autoimmune encephalitis, atypical Alzheimer’s disease). Diffusion-weighted imaging (DWI) is the most sensitive neuroimaging sequence for diagnosing CJD. However, early magnetic resonance imaging (MRI) findings may be subtle or evolving, and autoimmune etiologies often remain in the differential. Therefore, empiric corticosteroids are reserved for cases in which an autoimmune etiology is under consideration while definitive tests are pending. A 67-year-old woman presented with rapidly progressive cognitive decline, ataxia, and visual symptoms. Short-course glucocorticoids produced transient improvement for three days, followed by rapid deterioration within a week. Serial MRI evolved from cortical ribboning to basal ganglia involvement. Electroencephalogram (EEG) showed non-convulsive status epilepticus that responded to diazepam and valproate. Cerebrospinal fluid (CSF) 14-3-3 protein (14-3-3) and RT-QuIC were positive, confirming prion disease. CJD can present with features resembling HE, and brief improvement after a short course of glucocorticoids, even in the presence of markedly elevated thyroid antibodies, does not exclude CJD. To avoid diagnostic delay, obtain CSF RT-QuIC and 14-3-3 at presentation before or in parallel with glucocorticoids, and use serial MRI and EEG to arbitrate.

## 1 Background

Creutzfeldt–Jakob disease (CJD) is a uniformly fatal prion disease that presents with rapidly progressive dementia accompanied by myoclonus, visual or cerebellar dysfunction, extrapyramidal or pyramidal signs, and akinetic mutism [1], 2]. Diagnosis relies on clinical features supported by diffusion-weighted magnetic resonance imaging (MRI) showing cortical ribboning or basal ganglia hyperintensities, electroencephalogram (EEG) with periodic sharp-wave complexes, and cerebrospinal fluid (CSF) biomarkers such as RT-QuIC, 14-3-3 protein (14-3-3), and total tau [3]. Hashimoto’s encephalopathy (HE), also termed steroid-responsive encephalopathy associated with autoimmune thyroiditis (SREAT), is an autoimmune encephalopathy with subacute cognitive and psychiatric symptoms, seizures, and tremor or ataxia. MRI is often normal or nonspecific, EEG typically shows generalized slowing, CSF protein may be mildly elevated, and most patients improve with corticosteroids, IVIG, or plasma exchange [4], 5]. Misdiagnosis arises because CJD can mimic autoimmune encephalopathies [6] and antithyroid antibodies are prevalent and nonspecific, particularly in older adults; the diagnostic yield of a single early MRI or EEG may be limited and can improve on serial studies [7], 8]; CSF testing is often delayed due to its invasive nature and consent or logistical constraints; and limited access to RT-QuIC with slow turnaround encourages empiric immunosuppression while definitive results are pending. We report a patient with CJD who had elevated thyroid antibodies, transient cognitive improvement, and rapid deterioration after glucocorticoid pulse therapy, underscoring diagnostic pitfalls and the need to prioritize prion-specific biomarkers.

## 2 Case report

A 67-year-old woman was admitted to the hospital with a 2-week history of worsening neurological symptoms, including vertigo, blurred vision, diplopia, ataxia, and rapidly declining cognitive function. Four days before admission, she developed irritability and difficulty in expressing herself verbally. Her past medical, surgical, and family history was otherwise unremarkable, with no history of raw meat consumption, substance abuse, familial neurological disorders, neurosurgical procedures, blood transfusion, organ or tissue transplantation, or known exposure to individuals with similar symptoms. Detailed review of her residence history, dietary habits, and vaccination history did not reveal any clear source of prion exposure or infection.

On admission, her vital signs were stable, and general physical examination showed no abnormalities outside the nervous system. Routine laboratory tests, including complete blood count, serum electrolytes, liver and renal function tests, coagulation profile, and fasting glucose, were within normal limits. Screening for common infectious diseases (including hepatitis B and C, human immunodeficiency virus, and syphilis) was negative, and tumor markers were not elevated.

**Figure 1:**
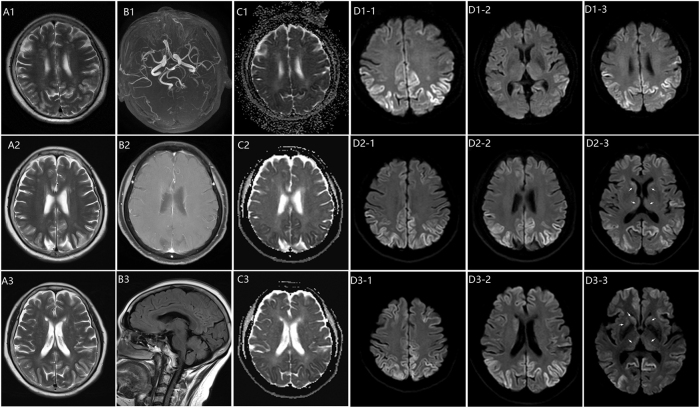
Serial brain MRI progression in prion disease. Images acquired on admission day 1 (top row), day 3 (middle row), and day 7 post-glucocorticoid (bottom row), across T2-weighted, MRA, ADC, and DWI sequences (left to right). Key DWI findings: day 1 – cortical ribbon sign with hyperintensity in bilateral temporoparietal and occipital cortices; day 3 – development of hockey stick sign in bilateral caudate heads and medial thalami (arrows); day 7 – new hyperintensity in the right globus pallidus (arrow) and more extensive cortical and deep gray matter involvement.

**Figure 2:**
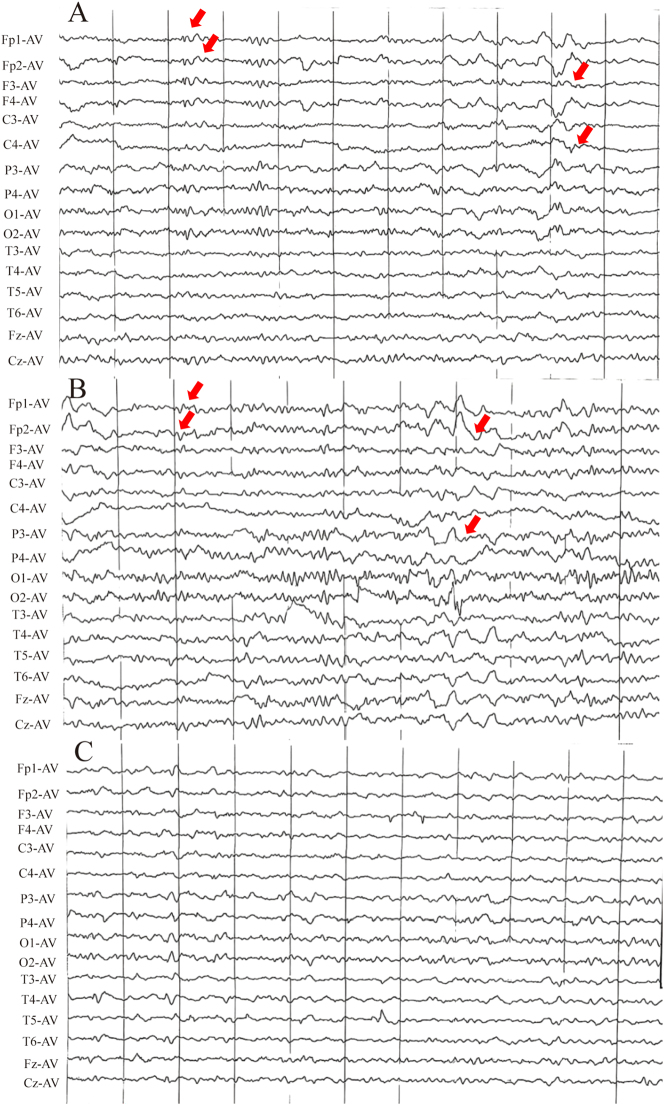
Serial EEG changes during hospitalization. (A) Day 1: slowed α background with mildly increased δ activity in all leads (arrows). (B) Day 3: after diazepam, normal α background with mildly increased medium-amplitude δ activity, most prominent over the right frontal and temporal regions (arrows). (C) Day 7: θ background after glucocorticoid therapy. EEG was recorded using an average reference (AV).

Gait impairment with rightward drift, recent memory loss, and tremors in the distal upper extremities was identified through a neurological examination upon admission. Muscle tone was normal, Romberg’s sign was positive, and bilateral Babinski and Hoffman signs were absent. Cognitive assessment revealed mild impairment, with a MoCA-BJ score of 17 [9]. Initial brain MRI demonstrated abnormal signals in the bilateral temporal, parietal, and occipital cortices, as well as the left frontal cortex and left semioval center (Figure 1). EEG indicated frontal slow waves (Figure 2A).

The patient exhibited an unsteady gait, perseverative behaviors, and thalamic aphasia, along with increased upper limb tremors and worsening cognitive function by the third day of hospitalization. Follow-up MRI revealed new abnormalities in the caudate nuclei and putamen (Figure 1: D2-3). Suspected non-convulsive status epilepticus (NCSE) was managed with intravenous diazepam and oral sodium valproate, resulting in temporary improvement (Figure 2).

The patient’s cognitive function declined significantly one week after admission. CSF analysis revealed normal cell counts, protein, and glucose levels, with no abnormalities. CSF findings are summarized in Supplementary Table S1. Thyroid ultrasonography showed diffuse thyroid enlargement, and serum anti-TPO and anti-TG antibodies were elevated. After endocrinology consultation, HE remained a leading consideration; therefore, a brief empiric course of high-dose glucocorticoids plus selenium yeast tablets was initiated while CSF prion studies were arranged, as CJD remained in the differential given diffusion-weighted imaging (DWI) cortical ribboning and concurrent NCSE. Despite initial improvement, the patient’s condition rapidly worsened seven days after starting glucocorticoid therapy. A comprehensive autoimmune panel, including serum and CSF autoantibodies (anti-NMDAR, anti-AMPAR, anti-GABAR, anti-LGI1, anti-CASPR2, and anti-aquaporin 4), returned negative results. Follow-up MRI showed progressive abnormalities (Figure 1: D3-3). CSF analysis by the Chinese CDC detected positive 14-3-3 protein, and RT-QuIC confirmed the presence of abnormal prion protein (PrP). Based on the clinical presentation, neuroimaging findings, and positive CSF biomarkers (14-3-3 protein and RT-QuIC), a definitive diagnosis of sporadic CJD was made. The patient succumbed 8 months after symptom onset. Postmortem neuropathological examination confirmed pathological prion protein (PrPSc), confirming the diagnosis of CJD.

## 3 Discussion

A patient diagnosed with sporadic Creutzfeldt-Jakob disease (sCJD) is presented in this case, characterized by rapid cognitive decline, cerebellar ataxia, visual impairment, and transient improvement of symptoms following glucocorticoid therapy. The presence of the ribbon sign on MRI, indicating cortical necrosis, contributed to the diagnosis.

Rapidly progressive cognitive decline requires a broad differential diagnosis including prion disease, rapidly progressive neurodegenerative dementia, autoimmune or infectious encephalitis, metabolic or toxic encephalopathy, vascular or inflammatory disease, and neoplastic involvement of the central nervous system. Huntington’s disease can also cause cognitive decline with psychiatric and behavioral symptoms [10], but usually follows a slow and insidious course rather than the subacute deterioration seen in our patient.

From a pathophysiological perspective, neurobehavioral manifestations in CJD are thought to result from dysfunction of large scale networks that include the frontal lobes, basal ganglia, and limbic system. Synaptic and mitochondrial impairment related to prion pathology in these regions may disturb cortical and subcortical connectivity as well as redox balance [11], and may underlie the rapid cognitive and behavioral decline in our patient.

The serial MRI findings in this case demonstrated the characteristic progression pattern of sCJD, offering crucial diagnostic evidence. The initial DWI sequences revealed cortical ribboning in the bilateral temporal, parietal, and occipital regions, which rapidly progressed to involve the basal ganglia within three days. This imaging evolution is consistent with recent studies, which demonstrates that DWI is the most sensitive neuroimaging sequence for diagnosing CJD, with cortical signal abnormalities detected in approximately 80–90 % of cases [8], 12]. The “cortical ribbon sign” observed represents restricted diffusion in the cerebral cortex, which has been established as an early and specific marker of CJD [8]. MRI revealed early, symmetrical hyperintensities in the basal ganglia, a pattern suggestive of prion-related pathology. Recent findings have demonstrated that basal ganglia involvement is present in approximately 73.4 % of CJD cases on DWI, compared to 37.8 % on FLAIR sequences [3]. The combination of cortical and basal ganglia involvement has demonstrated a sensitivity of 92 % and specificity of 80 % for diagnosing sCJD [8]. The imaging findings in this patient progressed from cortical ribboning to symmetrical basal ganglia hyperintensities, a pattern consistent with the temporal evolution reported in prion disease literature. The progressive nature of these changes, despite immunotherapy, further reinforced the diagnosis of CJD over other rapidly progressive dementias. Recent large cohorts and systematic reviews have shown that characteristic MRI abnormalities are present in roughly 80–90 % of patients with CJD, with a specificity in a similar 80–90 % range when distinguishing CJD from non-prion causes of rapidly progressive dementia [3], 8], 12].

The serial EEG recordings in this case revealed valuable diagnostic and monitoring features throughout the disease course. Initially, the EEG displayed nonspecific frontal slow waves, consistent with early-stage CJD, as reported by Hermann et al. [8]. Importantly, characteristic periodic sharp wave complexes (PSWCs) were not observed at any stage, even though such discharges are reported in roughly one-half to two-thirds of patients with sporadic CJD, particularly in more advanced phases of the disease [12], 13]. During the period of rapid neurological deterioration, the EEG instead showed patterns compatible with non-convulsive status epilepticus, with transient improvement after diazepam and sodium valproate, consistent with recent data indicating that seizures or seizure-like activity occur in a minority (approximately 15–20 %) of patients with CJD [12]. The absence of classic PSWCs in this case, despite a confirmed CJD diagnosis, supports the growing understanding that EEG patterns in CJD can be heterogeneous [14]. While EEG is a crucial diagnostic tool for CJD, this case highlights the need for careful interpretation taking into account the clinical context, disease stage, and potential confounding factors. It should also be integrated with other diagnostic methods for accurate diagnosis and management [15], 16]. Notably, peri-ictal cortical diffusion restriction associated with NCSE can mimic the cortical “ribbon sign” on DWI, which complicated early differentiation from autoimmune encephalopathy at presentation.

Interestingly, despite the progressive spread of abnormal MRI signals in the basal ganglia and cortex, a gradual resolution of sharp slow waves on the EEG was observed following the initiation of sodium valproate therapy, with no subsequent seizure manifestations. This therapeutic response indicates that the early recognition and prompt administration of antiepileptic medications in patients with probable CJD exhibiting seizure-like activities may be beneficial for symptom management. This observation aligns with recent studies, which indicate that timely anticonvulsant intervention may reduce seizure burden and improve the quality of life in patients with CJD, even as the underlying disease continues to progress [17]. This case underscores the significance of considering early antiepileptic treatment as part of the management strategy for probable patients with CJD presenting with seizure-like symptoms.

The definitive diagnosis of CJD in the case was supported by positive CSF 14-3-3 protein and RT-QuIC assay results. While 14-3-3 protein has traditionally served as a biomarker for CJD, with a sensitivity ranging from 85 % to 95 %, its specificity is relatively limited (60–75 %) due to its elevation in other neurological conditions [8]. In contrast, RT-QuIC has emerged as a groundbreaking diagnostic tool, demonstrating remarkable specificity (> 99 %) and sensitivity (92–96 %) for CJD diagnosis [18]. The combination of these biomarkers, especially the high specificity of RT-QuIC, facilitated rapid and accurate diagnosis of the patient. This is consistent with current diagnostic criteria, which highlight the importance of RT-QuIC as a dependable biomarker for ante-mortem CJD diagnosis [8], 12], 19]. In our setting, however, CSF 14-3-3 and RT-QuIC were available only as send-out tests (approximately 1-week turnaround), which delayed definitive confirmation at presentation.

CJD and HE can present with overlapping clinical features such as rapid cognitive decline, neuropsychiatric symptoms, and abnormal EEG findings - making misdiagnosis a critical concern. CJD, a fatal prion disease, along with positive CSF biomarkers like 14-3-3 protein and RT-QuIC [12]. In contrast, HE is an autoimmune encephalopathy associated with elevated anti-thyroid antibodies and often responds dramatically to corticosteroids [5]. Misdiagnosis arises when HE mimics CJD’s clinical trajectory but lacks definitive prion markers, leading to missed therapeutic windows. To prevent this, thyroid antibody testing, and CSF 14-3-3, RT-QuIC assays need to be included in the workup of rapidly progressive dementia, especially in middle-aged women, while consider a trial of high-dose glucocorticoid pulse therapy when autoimmune encephalopathy is suspected.

Due to abnormal thyroid-related antibody tests, HE was suspected, and glucocorticoid pulse therapy was administered. This decision balanced the differential while send-out prion testing was pending, with close MRI, EEG follow-up. While the patient exhibited initial improvement within 7 days of treatment, likely due to a reduction in neuroinflammation, the subsequent clinical course was characterized by dramatic deterioration, accompanied by newly developed abnormal signals in the right lentiform nucleus on the third MRI. This pattern, though differing, shares key similarities with the case reported by Jang et al., where a patient with CJD presenting with features mimicking HE demonstrated immediate deterioration following glucocorticoid pulse therapy [20]. The eventual rapid decline in the patient, despite the initial response, raises concerns about the potential role of glucocorticoids in accelerating CJD progression. This observation underscores the importance of excluding CJD through CSF 14-3-3 protein and RT-QuIC testing before initiating steroid therapy in suspected HE cases, particularly those presenting with rapid progressive dementia [3], 21].

Additionally, our investigation of the patient’s residence history, dietary habits, and vaccination history revealed no clear source of infection, and our patient had no documented SARS-CoV-2 infection. Recent reports suggest that long COVID may present with neurological symptoms and MRI findings overlapping with prion diseases [22], 23]. Therefore, asymptomatic prior infection cannot be entirely excluded.

## 4 Conclusions

CJD can present with features resembling HE, and brief improvement after a short course of glucocorticoids, even in the presence of markedly elevated thyroid antibodies, does not exclude CJD; the rapid deterioration after steroids observed in this case and in a prior Korean report raises concern for possible acceleration, although causality cannot be established. To avoid diagnostic delay, obtain CSF RT-QuIC and 14-3-3 at presentation before or in parallel with glucocorticoids, and use serial MRI and EEG to arbitrate.

## Supplementary Material

Supplementary Material
