# Supplementary material for: Creutzfeldt-Jakob disease mimicking Hashimoto’s encephalopathy: steroid response followed by decline
Source: Open Life Sci. 2025 Dec 30;20(1):20251245. doi: 10.1515/biol-2025-1245 (PMC13011900; doi:10.1515/biol-2025-1245)
Supplement: Supplementary file 1 — Supplementary Material [file j_biol-2025-1245_suppl_001.docx]

**Supplementary Table S1.** Cerebrospinal fluid results

| Category | Test | Method | Result | Reference range | Unit | Notes |
| --- | --- | --- | --- | --- | --- | --- |
| Routine/cytology | Appearance | Visual | Colorless, clear | — | — | — |
|  | Clarity | Visual | Clear | — | — | — |
|  | Pandy test | Chemical | Positive | Negative | — | — |
|  | White blood cell count | Cell count | 8 | 0–8 | ×10^6^/L | — |
|  | Red blood cell count | Cell count | 0 | — | ×10^6^/L | — |
| Biochemistry | Total protein | Biochemistry | 0.392 | 0.15–0.45 | g/L | — |
|  | Lactate dehydrogenase (LDH) | Biochemistry | 16 | 8–50 | U/L | — |
|  | Adenosine deaminase (ADA) | Biochemistry | 2 | 0–5 | U/L | — |
|  | Glucose | Biochemistry | 3.87 | 2.5–4.5 | mmol/L | — |
|  | Chloride | Biochemistry | 124.9 | 123–130 | mmol/L | — |
| Microbiology | Bacterial culture | Culture (7 days) | No growth | Negative | — | — |
|  | Cryptococcal capsular antigen | Antigen test | Negative | Negative | — | — |
| mNGS | Bacteria/fungi/parasites/DNA viruses | IDseq (DNA workflow) | Not detected | Negative | — | RNA viruses are not covered by this DNA workflow |
| mNGS (special pathogens) | Mycobacterium tuberculosis complex, NTM, Mycoplasma/Chlamydia | IDseq (DNA workflow) | Not detected | Negative | — | — |
| Autoimmune | Autoimmune encephalitis antibody panel: NMDAR, LGI1, CASPR2, GABA_BR, AMPAR1, AMPAR2, GAD65, MOG | CBA | All negative | Negative | — | — |
|  | Tissue‑based assay (cerebellum, hippocampus, other brain tissue) | TBA | Negative | Negative | — | — |
| Thyroid‑related antibodies (CSF) | TSH receptor antibody (TRAb) | RIA | <0.80 | 0–1.75 | IU/L | — |
|  | Anti‑thyroglobulin antibody (TgAb) | CLIA | <15.00 | 0–60 | IU/mL | — |
|  | Anti‑thyroid peroxidase antibody (TPOAb) | CLIA | <28.00 | 0–60 | IU/mL | — |
| Prion biomarkers | 14‑3‑3 protein | Immunoblot | Positive | Negative | — | — |
|  | RT‑QuIC | RT‑QuIC | Positive | Negative | — | — |

All results derive from the same CSF specimen. Reference ranges are those of the local laboratory. Abbreviations: CBA, cell‑based assay; TBA, tissue‑based assay; RT‑QuIC, real‑time quaking‑induced conversion; NTM, nontuberculous mycobacteria.
